# Supplementary material for: Mechanisms governing the pioneering and redistribution capabilities of the non-classical pioneer PU.1
Source: Nat Commun. 2020 Jan 21;11:402. doi: 10.1038/s41467-019-13960-2 (PMC6972792; doi:10.1038/s41467-019-13960-2)
Supplement: Supplementary file 7 — Source data [file 41467_2019_13960_MOESM7_ESM.zip › Source_Data/FigureS3/FigureS3J_MotifScanOutput/homerResults/motif31.similar.html]

motif31

## Information for motif31

A
C
G
T
C
A
T
G
C
G
T
A
G
A
C
T
C
T
A
G
G
T
C
A
C
G
T
A
C
G
T
A
A
G
C
T
G
A
T
C
C
G
A
T
G
A
T
C
A
T
G
C
  
Reverse Opposite:  

T
A
C
G
C
T
A
G
G
C
T
A
C
T
A
G
C
T
G
A
A
C
G
T
A
C
G
T
A
C
G
T
A
G
T
C
C
T
G
A
C
G
A
T
G
T
A
C
G
T
C
A
  

|  |  |
| --- | --- |
| p-value: | 1e-40 |
| log p-value: | -9.265e+01 |
| Information Content per bp: | 1.778 |
| Number of Target Sequences with motif | 111.0 |
| Percentage of Target Sequences with motif | 0.84% |
| Number of Background Sequences with motif | 25.9 |
| Percentage of Background Sequences with motif | 0.07% |
| Average Position of motif in Targets | 310.6 +/- 253.9bp |
| Average Position of motif in Background | 308.9 +/- 199.8bp |
| Strand Bias (log2 ratio + to - strand density) | 0.1 |
| Multiplicity (# of sites on avg that occur together) | 1.00 |
| Motif File: | file (matrix) reverse opposite |

### Similar de novo motifs found

|  |  |  |  |  |  |  |  |
| --- | --- | --- | --- | --- | --- | --- | --- |
| Rank | Match Score | Redundant Motif | P-value | log P-value | % of Targets | % of Background | Motif file |
| 1 | 0.872 | C A T G A G C T T C G A G A C T T C A G C T G A C A G T C T A G G T C A G T C A G T C A A C G T G A T C G C A T | 1e-38 | -88.283863 | 0.74% | 0.05% | motif file (matrix) |
| 2 | 0.918 | G T C A C T A G C G T A A C G T A C G T A C G T A G T C C G T A A C G T A G T C C G T A | 1e-33 | -76.280180 | 1.12% | 0.22% | motif file (matrix) |
| 3 | 0.953 | A G C T C T A G C T G A C G A T C T A G C T G A G T C A G T C A C G A T T G A C C G A T G A T C | 1e-29 | -68.918452 | 0.60% | 0.05% | motif file (matrix) |
| 4 | 0.809 | A C T G C G T A A G C T A G C T A C G T A G T C C T A G A C G T A G T C C T G A | 1e-28 | -65.004672 | 1.15% | 0.28% | motif file (matrix) |
